# Changes in Small Airway Physiology Measured by Impulse Oscillometry in Subjects with Allergic Asthma Following Methacholine and Inhaled Allergen Challenge

**DOI:** 10.3390/jcm14030906

**Published:** 2025-01-30

**Authors:** Henning Stenberg, Rory Chan, Khalid Abd-Elaziz, Arjen Pelgröm, Karin Lammering, Gerda Kuijper-De Haan, Els Weersink, René Lutter, Aeilko H. Zwinderman, Frans de Jongh, Zuzana Diamant

**Affiliations:** 1 Center for Primary Health Care Research, Department of Clinical Sciences, Malmö, Lund University, 21428 Malmö, Sweden; henning.stenberg@med.lu.se; 2 University Clinic Primary Care Skåne, 29189 Kristianstad, Region Skåne, Sweden; 3 School of Medicine, University of Dundee, Dundee DD1 9SY, UK; r.chan@dundee.ac.uk; 4 Department of Clinical Pharmacy and Pharmacology, University Medical Centre Groningen, 9700 RB Groningen, The Netherlands; k.s.abd-elaziz@umcg.nl; 5 Department of Pulmonary Medicine, Amsterdam University Medical Centre, 1007 MB Amsterdam, The Netherlands; a.t.pelgrom@amsterdamumc.nl (A.P.); e.j.weersink@amsterdamumc.nl (E.W.); r.lutter@amsterdamumc.nl (R.L.); f.h.dejongh@amsterdamumc.nl (F.d.J.); 6 Lung Function Centre O_2_CO_2_, 2582 EZ The Hague, The Netherlands; karinlammering@gmail.com; 7 QPS-NL, 9713 AG Groningen, The Netherlands; gerda.kuijperdehaan@gmail.com; 8 Department of Epidemiology and Data Sciences, Amsterdam University Medical Centre, 1105 AZ Amsterdam, The Netherlands; a.h.zwinderman@amsterdamumc.nl; 9 Department of Microbiology Immunology & Transplantation, Catholic University of Leuven, 3000 Leuven, Belgium; 10 Department of Respiratory Medicine, First Faculty of Medicine, Thomayer Hospital, Charles University, 12108 Prague, Czech Republic

**Keywords:** asthma, small airways, impulse oscillometry, lung function, methacholine challenge, allergen bronchoprovocation test

## Abstract

**Background**: Small airway dysfunction (SAD) is associated with impaired asthma control, but small airway physiology is not routinely assessed in clinical practice. Previously, we demonstrated impulse oscillometry (IOS)-defined small airway dysfunction (SAD) in dual responders (DRs) upon bronchoprovocation with various allergens. **Aim**: To compare lung physiology using spirometry and IOS following bronchoprovocation with methacholine (M) and inhaled house dust mite (HDM) extract in corticosteroid-naïve asthmatic subjects. **Methods**: Non-smoking, clinically stable HDM-allergic asthmatic subjects (18–55 years, FEV_1_ > 70% of pred.) underwent an M and inhaled HDM challenge on two separate days. Airway response was measured by IOS and spirometry, until a drop in FEV_1_ ≥ 20% (PC_20_) from post-diluent baseline (M), and up to 8 h post-allergen (HDM). Early (EAR) and late asthmatic response (LAR) to HDM were defined as ≥20% and ≥15% fall in FEV_1_ from post-diluent baseline during 0–3 h and 3–8 h post-challenge, respectively. IOS parameters (Rrs5, Rrs20, Rrs5-20, Xrs5, AX, Fres) were compared between mono-responders (MRs: EAR only) and dual responders (EAR + LAR). Correlations between maximal % change from baseline after the two airway challenges were calculated for both FEV_1_ and IOS parameters. **Results**: A total of 47 subjects were included (11 MRs; 36 DRs). FEV_1_ % predicted did not differ between MR and DR at baseline, but DR had lower median PC_20_M (0.84 (range 0.07–7.51) vs. MR (2.15 (0.53–11.29)); *p* = 0.036). During the LAR, DRs had higher IOS values than MRs. For IOS parameters (but not for FEV_1_), the maximal % change from baseline following M and HDM challenge were correlated. PC_20_M was inversely correlated with the % change in FEV_1_ and the % change in Xrs5 during the LAR (r= −0.443; *p* = 0.0018 and r= −0.389; *p* = 0.0075, respectively). **Conclusions**: During HDM-induced LAR, changes in small airway physiology can be non-invasively detected with IOS and are associated with increased airway hyperresponsiveness and changes in small airway physiology during methacholine challenge. DRs have a small airways phenotype, which reflects a more advanced airway disease.

## 1. Introduction

The small airways are usually defined as the distal branches of the bronchial tree with an internal diameter of less than 2 mm and represent the so-called “silent zone”, usually unattended in daily practice [1]. Nevertheless, small airways dysfunction (SAD) is considered an important feature of asthma, and associations have been demonstrated with nocturnal symptoms, impaired asthma control, increased exacerbation rates and exercise-induced bronchoconstriction [2]. The small airways are also the site of remodeling across all asthma severities, ranging from mild to fatal [3,4]. During exacerbations, impact on small airways has been reported by various studies, e.g., with changes in residual volume, increased levels of alveolar nitric oxide (NO) as well as bronchial wall thickening and air trapping assessed by computed tomography [2,5]. However, there is still debate on how best to assess SAD in asthma, on what some of the parameters retrieved by the different methods actually represent (e.g., with doubts regarding how much the pathophysiology of large airways and ventilation heterogeneity contribute) and uncertainties about reference equations for normal values of numerous parameters. Assessments of small airway function are, therefore, presently not included in guidelines nor in clinical practice, despite oscillometry demonstrating usefulness in the diagnosis and management of asthma [6,7]. Furthermore, although the effects of direct stimuli on distal airways have been extensively studied, there is still a knowledge gap in understanding the pathophysiology of the small airways following different (direct and indirect) stimuli [8,9,10,11,12].

The allergen bronchoprovocation test is a standardized test allowing the study of the pathophysiology and underlying mechanisms following allergen inhalation as well as a valued tool in proof-of-concept studies with new interventions [13,14]. Inhalation of a relevant allergen induces an early asthmatic response (EAR) within the airways of sensitized asthmatic subjects, which is followed by a late asthmatic response (LAR) in approximately 50% of individuals [13,15]. The LAR is characterized by airway inflammation, which produces longer-lasting airflow obstruction in both the large and the small airways, and is known to increase nonspecific airway hyperresponsiveness (AHR) [12] and possess pro-remodeling activity [16]. The EAR occurs within 10–20 min upon inhalation of the provocative allergen dose, subsides within approximately 1–3 h and is primarily bronchospastic. While the EAR is likely more associated with proximal airways pathophysiology, the LAR has been shown to produce pathophysiological changes measured by several different methods that are theoretically connected to distal airways [17,18]. Although allergen challenge is mainly an investigational tool [13,14], the differentiation between mono (EAR only) and dual (EAR + LAR) responder types may have clinical implications, particularly in patient phenotyping and tailoring of therapeutic strategies [19].

Impulse oscillometry (IOS) is a sensitive, non-invasive technique that allows assessment of airway resistance and reactance, thus providing insights into the function, supposedly within both the central and the small airways, which can optimize and support diagnostic, therapeutic and monitoring purposes [20,21,22]. The IOS technique applies small flow oscillations at the mouth to measure the mechanical properties of the central and distal airways as well as the lung tissue. Oscillations of lower frequencies (5 Hz) will reach more distal airways, while the higher frequencies (20 Hz) will only reach the larger proximal airways. The pulmonary impedance (i.e., forces that hinder airflow into and out of the lung) that can be measured with IOS is composed of resistance (Rrs) and reactance (Xrs). The resistance is dependent on airway caliber and is calculated from the flow that arises from a certain pressure. The reactance, however, is a more complex concept associated with both inert and elastic forces, i.e., the airflow that is “out of phase” with the oscillations. Simply put, inertance is the opposing force building up before the flow, while elastance is the lagging force recoiling after the flow. The reactance to low-frequency oscillations (Xrs5) is more relevant to the elastic forces of the lung tissue (also affected by bronchoconstriction), while reactance to higher frequencies is more affected by the inert forces (mainly the column of air within the airways) and has little clinical importance. By convention, elastic forces are negative in sign while inertance is positive. The frequency where elastic and inert forces are equal (i.e., where reactance is zero) is called the resonant frequency (Fres), and the integrated area of reactance from Fres to Xrs5 is called AX. With increased “stiffness” of the lungs (e.g., due to bronchoconstriction, airway edema or remodeling), Fres and AX will increase.

In this observational study, we investigated the difference and relationship between airway responses of the large airways (mainly reflected by FEV_1_) and the small airways (assessed by IOS) to methacholine (M; direct stimulus) and inhaled allergen (HDM; indirect stimulus). In addition, we compared the difference in airway physiology parameters between mono (MRs) versus dual responders (DRs) to inhaled allergen challenge.

## 2. Methods

### 2.1. Subjects and Study Design

Data were derived from the screening phase of a 2-center phase 2A study sponsored by Foresee Pharmaceuticals (Taipei, Taiwan) that started in May 2018, with the last patient concluded in May 2022. The study was registered in www.clinicaltrials.gov (NCT03858686, accessed on 6 January 2025). Participants were subjects (F/M; 18–55 years) with clinically stable mild asthma without any asthma medication or only using short-acting beta_2_ agonists (SABA) on an infrequent, *prn* basis. Asthma diagnosis was established according to GINA 2018 guidelines [23]. Main inclusion criteria were in line with the usual prerequisites for an inhaled allergen challenge [13] and comprised physician-established asthma diagnosis, forced expiratory volume in the first second (FEV_1_) ≥ 70% predicted, methacholine provocative concentration causing a 20% fall in FEV_1_ (PC_20_ methacholine chloride) < 16 mg/mL; i.e., PC_20_ methacholine bromide < 19.6 mg/mL), blood eosinophil count ≥ 150 cells/µL and confirmed house dust mite (HDM) allergy. Major exclusion criteria included current or history of clinically relevant disease other than asthma, respiratory (viral) infections including common cold and COVID-19 within 3 weeks of screening, current smoker or ex-smoker (within 12 months of study start and ≥5 pack-years; current non-smoking was confirmed by a negative cotinine rapid test), pregnancy or lactation, abuse of alcohol or drugs, the use of asthma maintenance medications including allergen specific immunotherapy, biologics, inhaled or systemic corticosteroids or any study medication [13].

The study was approved by an independent ethics committee (Stichting BeBo, Assen, The Netherlands, approval date: 22 May 2018, CCMO code: NL64799.056.18), and all subjects provided written informed consent prior to participation.

The screening was performed on 3 separate days. The main aim of the first screening visit (S1) was to obtain informed consent, confirm subject eligibility based on inclusion and exclusion criteria and to confirm HDM allergy (by blood (ImmunoCap Phadiatop (ThermoFisher Scientific, Eindhoven, The Netherlands)) or skin prick test (ALK-Abello, Almere, The Netherlands)).

On screening visit 2 (S2), a standard methacholine challenge was performed until an FEV_1_ drop ≥ 20% from post-diluent baseline was obtained to calculate the PC_20_ (by interpolation) with IOS measurements preceding spirometry. During the worldwide shortage of supplies, instead of methacholine, histamine was used in 3 DRs (as detailed below).

During screening visit 3 (S3), a standardized allergen challenge was performed with inhaled HDM extract (HAL Allergy, Leiden, The Netherlands), and the FEV_1_ response (preceded by IOS) was measured during the EAR (0–3 h) at 10, 20, 30, 45, 60, 90, 120 and 240 min and subsequently at hourly intervals during the LAR (3–8 h) until 8 h post-challenge or 9 h if deemed necessary by the investigator (to confirm LAR criteria).

### 2.2. Study-Related Procedures

All study procedures, assessments and equipment used in the study were identical across both study centers and all measurements were conducted at the same time of the day (±2 h) in each participant.

### 2.3. Spirometry

Lung function tests were performed with a regularly serviced and calibrated Masterscreen Pneumo spirometer (Vyaire, Mettawa, IL, USA), according to current standards of the European Respiratory Society (ERS)/American Thoracic Society (ATS) [24]. Spirometry was measured at baseline and at preset timepoints during bronchoprovocation tests with methacholine/histamine and inhaled HDM allergen.

### 2.4. Impulse Oscillometry (IOS)

Airway resistance, impedance and reactance were measured using Vyntus Impulse oscillometry (IOS) equipment (Vyaire, Mettawa, IL, USA), according to standard methodology [25]. Oscillometric pressure impulses with a frequency spectrum between 5 and 35 Hz were applied during tidal breathing for at least 30 s. Mean values of resistance at 5 Hz (Rrs5) and 20 Hz (Rrs20), reactance at 5 Hz (Xrs5), integrated area of reactance from Fres to Xrs5 (AX) and resonant frequency (Fres) were assessed. Rrs5 minus Rrs20 was calculated to determine the frequency dependence of resistance (Rrs5-20) and Rrs5-20 corrected for Rrs5 was also calculated (Rrs5-20/Rrs5) [26]. IOS was performed prior to spirometry, before the respective challenges (baseline) and after each dosing step (M) or at each preset timepoint post-allergen (HDM).

### 2.5. Methacholine (and Histamine) Bronchoprovocation Test

Methacholine challenge (methacholine bromide, A15 Apotheek, Gorinchem, The Netherlands) was performed according to the guidelines [27,28]. Following inhalation of the diluent (NaCl 0.9%), incremental (doubling) dose steps of methacholine bromide (0.038–19.6 mg/mL) or histamine sulphate (0.03–16 mg/mL) were nebulized by a calibrated (0.13 mL/min) jet-nebulizer (DeVilbiss Healthcare GmbH, Mannheim, Germany) at ±5 min intervals and inhaled for 2 min by tidal breathing, as previously described [29]. After each dose step, airway response was measured in duplicate approximately 30 and 90 s post-dose, and the highest, technically satisfactory value was included in the analysis and expressed as % change from post-diluent baseline. IOS measurements were performed prior to spirometric measurements to minimize the change in bronchomotor tonus caused by spirometry. The test was concluded until FEV_1_ dropped ≥20% from post-diluent baseline and the PC_20_FEV_1_ (methacholine) or PC_20_FEV_1_ (histamine) was calculated by interpolation [30]. After the challenge, subjects were given salbutamol to aid recovery.

Due to shortages and supply disruptions caused by the COVID-19 pandemic and Brexit, methacholine bromide was replaced by histamine (H) sulphate (A15 Apotheek, Gorinchem, The Netherlands) in 3 subjects (all of them DRs). Although interacting with different receptors; both histamine and methacholine are regarded as direct agonists producing bronchoconstriction, as outlined in the international guidelines by ATS/ERS; both have shown to yield similar outcomes in terms of PC_20_ as well as in IOS parameters [29,31]; and both have been (interchangeably) used to assess nonspecific airway hyperresponsiveness according to the same protocol in standard clinical practice [29] as well as in the context of the allergen bronchoprovocation test [29,32]. Furthermore, both histamine (which was historically the first agent) and methacholine have been used to predict the allergen PC_20_ in a similar way [32,33]. The dose steps for histamine sulphate and methacholine chloride are identical, but a conversion factor of 1.23 is usually applied from histamine sulphate/methacholine chloride (with a molecular weight of 195.69 g/mol) to methacholine bromide (with a molecular weight of 240.14 g/mol) [34].

### 2.6. Allergen Bronchoprovocation Test

Allergen challenge was conducted using a standardized technique with incremental doubling dilutions of aqueous *Dermatophagoides pteronyssinus* extract (HAL allergy, Leiden, The Netherlands) [13]. Within 24 h before S3, serial doubling dilutions ranging from 5000 to 5 allergy units (AU)/mL were prepared from the stock solution in diluent (disodium phosphate dihydrate, sodium dihydrogen phosphate dihydrate, aminocaproic acid, human serum albumin, glycerol and phenol in water for injection or normal saline). The predicted provocative dose of inhaled HDM extract was calculated according to the modified formula by Cockcroft et al. [13,32,33]. Each allergen challenge was preceded by inhalation of the diluent and, provided the post-diluent FEV_1_ remained within 10% of the pre-diluent value, incremental doubling concentrations of HDM extract were aerosolized by a DeVilbiss 646 jet-nebulizer (output 0.13 mL/min) at approximately 12 min intervals, and each concentration was inhaled by tidal breathing for 2 min, through the mouth with the nose clipped. Airway response to inhaled HDM was measured in duplicate 10 min following each concentration until a drop in FEV1 ≥ 20% from the post-diluent value was reached and consequently at 20, 30, 45, 60, 90, 120 min and at hourly intervals until 8 h post-allergen (or 9 h post allergen if deemed necessary and safe by the investigator). At each timepoint, the highest, technically satisfactory value was expressed as % change from post-diluent FEV_1_ and included in analysis. IOS measurements (Rrs5, Rrs20, Xrs5, AX, Fres) were performed prior to the spirometric measurements at each timepoint. After completion of the test, subjects were provided salbutamol, oral and written instructions as well as emergency phone numbers and could be sent home from the unit if clinically stable.

### 2.7. Statistical Analysis

Demographic characteristics and baseline lung function parameters (i.e., before the methacholine/histamine and HDM challenges) were summarized for MRs and DRs using median values (minimum, maximum) for quantitative variables and proportions of categorical variables, and compared using nonparametric and chi-square tests.

Mean % changes in FEV_1_ and IOS parameters, as a function of doubling methacholine/histamine doses and as a function of time post-HDM challenge, were illustrated graphically together with pointwise 95% confidence intervals for both MRs and DRs. In addition, the differences in the mean % changes in the FEV_1_ and IOS parameters between MRs and DRs were plotted together with the pointwise 95% confidence intervals. When the confidence intervals of the mean differences excluded the value zero, this was interpreted as an indication of a significant difference between MRs and DRs. We also fitted mixed-effects models of the change in the IOS parameters, with or without main-effect of the MR versus DR variables and their interaction with dose and time, to test statistical differences between MRs and DRs. The non-parametric Spearman’s correlation coefficient was used for the calculations of correlations between maximal % change in physiology parameters, and between methacholine/histamine PC_20_ and maximal % change in physiology parameters post-allergen.

## 3. Results

### 3.1. Subjects

Complete data sets (S1–S3) from 47 participants were available, with 11 and 36 subjects fulfilling definitions for MR and DR, respectively. Three subjects (all DRs) received histamine at S2 while the rest of the subjects received methacholine bromide. Table 1 summarizes the subject demographics. Compared to MRs, DRs had a higher mean baseline Rrs5 at visit S2 (pre-methacholine/histamine challenge) (0.27 (0.17–0.41) vs. 0.36 (0.21–0.66) kPa/L/s, *p* = 0.012) and lower mean methacholine/histamine PC_20_ (2.15 (0.53–11.29 vs. 0.84 (0.07–7.51); *p* = 0.036) (Table 1). In DRs, a non-significant trend was observed towards a lower mean baseline FEV_1_ (*p* = 0.11), Rrs5-20 (*p* = 0.09) and AX (*p* = 0.088) at visit S2.

### 3.2. Methacholine/Histamine Challenge

**Figure 1:**
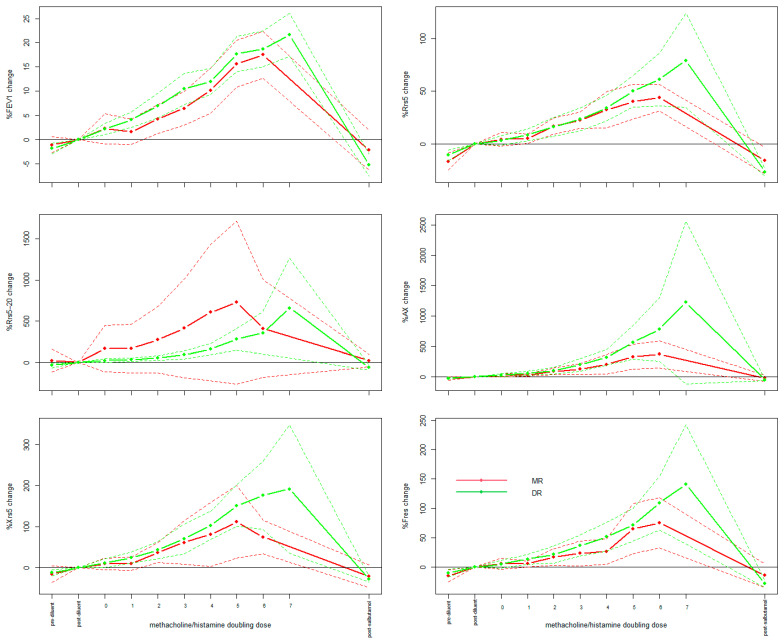
Mean % change from post-diluent baseline of spirometry and oscillometry measurements in response to methacholine/histamine at screening visit 2 (S2). Data shown for MR (red) and DR (green); solid lines are mean values; dotted lines refer to 95%CIs.

At visit S2, no significant differences were detected in the % changes in FEV_1_, Rrs5, Rrs5-20, Rrs5-20/R5, AX, Xrs5 or Fres comparing MRs and DRs at any doubling dose of methacholine/histamine (Figure 1).

### 3.3. Inhaled Allergen Challenge

**Figure 2:**
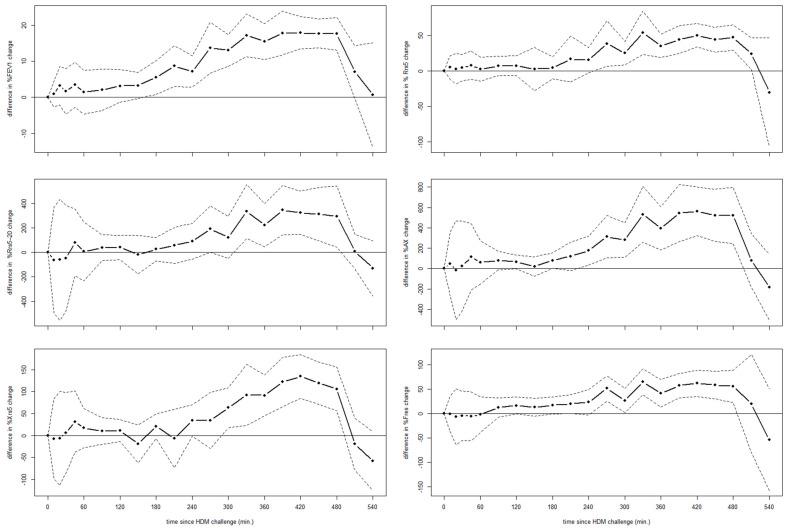
Mean differences in % change of spirometry and oscillometry values comparing DR versus MR to HDM challenge over time. Dotted lines refer to 95%CIs.

At visit S3, the % changes in FEV_1_, Rrs5, Rrs5-20, AX, Xrs5 and Fres during the LAR (3–8 h post-allergen) were significantly larger (*p* < 0.0001, all parameters) in DRs compared to MRs (Figure 2). No statistically significant differences in any of these parameters were observed comparing MRs and DRs during the EAR (0–3 h post-allergen).

## 4. Correlations

Table 2 shows the correlation coefficients for the maximal % changes (from post-diluent baseline) in FEV_1_ and in six of the IOS parameters (i.e., Rrs5, Rrs5-20, Rrs5-20/Rrs5, X5, AX and Fres), comparing airway response following methacholine/histamine challenge as well as during the allergen-induced EAR and the LAR. Statistically significant correlations were found for all the IOS parameters (Rrs5, Rrs5-20, Rrs5-20/Rrs5, X5, AX and Fres) when comparing the maximal response after methacholine/histamine challenge to the maximal response during the EAR (0–3 h post-allergen). This was not significant for FEV_1_. As for correlations between the maximal % change after methacholine/histamine challenge and during the LAR (3–8 h post-allergen), Rrs5, Rrs5-20, Rrs5-20/Rrs5, AX and Fres were significantly correlated, while FEV_1_ and Xrs5 were not. Comparing the EAR to the LAR, Rrs5-20, Rrs5-20/Rrs5, AX and Fres were significantly correlated, while FEV_1_, Rrs5 and Xrs5 were not correlated.

**Figure 3:**
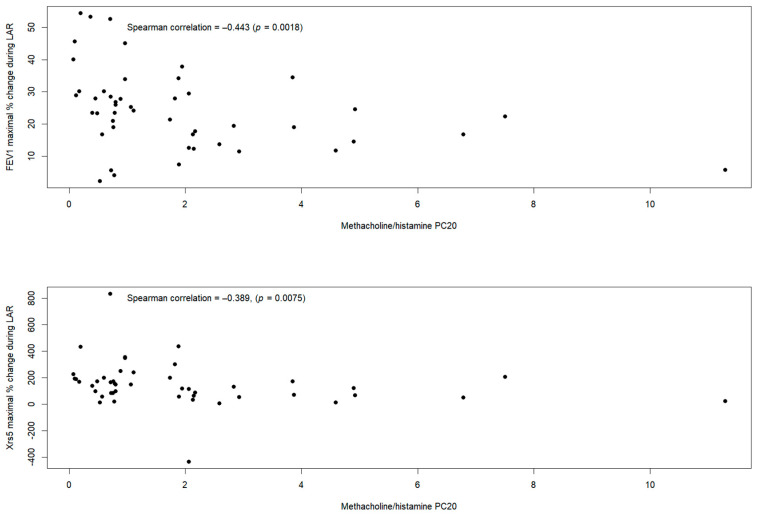
Association between methacholine/histamine PC_20_ and maximal % change in FEV_1_ (**upper panel**) and Xrs5 (**lower panel**) during the LAR.

The methacholine/histamine PC_20_ was inversely and significantly correlated with the % change in FEV_1_ during the LAR (3–8 h post-allergen) (r = −0.443; *p* = 0.0018) and with the % change in Xrs5 during the LAR (3–8 h post-allergen) (r = −0.389; *p* = 0.0075) (Figure 3). No correlations were observed between methacholine/histamine PC_20_ and % change in FEV_1_ during the EAR or any of the other IOS parameters (Rrs5, Rrs5-20, Rrs5-20/Rrs5, AX or Fres) expressed as maximal % change from baseline during the EAR or the LAR.

## 5. Discussion

### 5.1. Summary of Study Findings

Here, we report changes in airway physiology after both a direct (methacholine/histamine) and an indirect (inhaled allergen; HDM) challenge in non-smoking subjects with HDM-allergic, eosinophilic, mild asthma not on controller therapy. In this study, we investigated parameters that reflect the large airways as well as those that are thought to represent the small airways and compared the airway responses between MRs and DRs to inhaled HDM. We observed that at baseline, DRs presented with overall lower methacholine/histamine PC_20_ and a non-significant trend towards more compromised proximal and distal lung function, indicative of more advanced airway disease. During the allergen-induced LAR (3–8 h post-allergen), both the large airway parameters (measured by spirometry: FEV_1_) and the small airway parameters measured by IOS (Rrs5-20, Xrs5 and AX) were more affected in DR compared to MR. In addition, significant correlations were observed between the changes in small airway parameters following the two different types of airway challenges, indicating that some asthmatics tend to respond with more peripheral airway obstruction regardless of the type of challenge. Furthermore, we found inverse (negative) correlations between methacholine/histamine PC_20_ and small airway reactance, indicating that subjects with increased AHR have a greater peripheral lung stiffness, which may consist of SAD and airway remodeling [35]. Although the pathophysiology of asthma is complex [36], we opted to keep our study focused on physiological parameters related to the allergy response and SAD.

### 5.2. Comparison with Existing Literature

Our findings of changes in large and small airway physiology following both a methacholine/histamine challenge and an inhaled allergen challenge confirm and extend previous studies [18,37]. Here, we show that a higher degree of non-specific AHR, i.e., low methacholine/histamine PC_20_, is associated with HDM-induced LAR with increased small airway reactance in subjects with HDM-allergic asthma. Our findings clearly demonstrate the interrelationship between AHR [38], the allergen-induced LAR (which represents a prototype of type 2 allergic eosinophilic airway inflammation [39]) and small airway disease, which reflects aspects of airway remodeling [40]. From a clinical perspective, this underscores the need to proactively identify and treat airway hyperresponsiveness/inflammation with anti-inflammatory, disease-modifying therapies, even in asymptomatic patients.

Indeed, in contrast to the EAR, the LAR is closely associated with type 2 inflammation, enhanced AHR and airway remodeling, which are related to SAD [18,41,42,43]. Our study, therefore, raises the interesting question as to whether targeted biologics (omalizumab, dupilumab, tezepelumab) and/or allergen immunotherapy (AIT) would suppress SAD owing to their systemic anti-inflammatory properties and disease-modifying potential [44]. Previously, systemic use of anti-IgE monoclonal antibody (rhuMAb-E25; later licensed as omalizumab) in individuals with allergic asthma has been shown to significantly reduce serum IgE as well as the LAR (vs. placebo) [43,45]. Whilst it is unknown whether omalizumab improves oscillometry measurements, it has been shown to improve FEF_25–75_, [46], which several researchers have considered as a measure of small airways [47]. However, doubts have also arisen whether FEF_25–75_ is really a useful and independent marker of small airway function, as it is highly variable and dependent on FEV_1_ [48]. Blocking thymic stromal lymphopoietin (TSLP) with tezepelumab reduced serum total IgE levels, improved FEF_25–75_ as well as attenuated the maximum % fall in FEV_1_ during the LAR in individuals with mild allergic asthma [49,50,51]. Similarly, treatment with HDM subcutaneous (SCIT) and sublingual (SLIT) immunotherapy improved symptoms and lung function in individuals with allergic asthma, while several studies attested to the efficacy of allergen immunotherapy (AIT) in attenuating the allergen-induced LAR [52,53,54]. Anti-IL4Rα dupilumab is another monoclonal antibody that simultaneously modified allergic inflammation and improved Rrs5-20 and AX [55,56], although it is unknown whether it also attenuates allergen-induced LAR.

The small airways have previously been termed the quiet zone of the lungs due to the difficulty experienced by clinicians treating conditions that affect this area [57]. Inhaled corticosteroids are excellent topical anti-inflammatories but are limited by their larger particle sizes that end up being deposited in the large airways and oropharynx. The data on extra-fine ICS is also not particularly robust. Based on this, one might expect systemic forms of therapy including biologics and AIT to bypass this problem. We hope the present study will generate more interest in this area.

To our knowledge, it is a novel finding that the degree of small airway pathophysiological changes is significantly correlated when comparing two different types of airway challenges. This further supports the notion that some asthmatics, especially DRs, represent a “small airways phenotype”, with a certain set of clinical features associated with it [2]. More studies are needed to investigate whether this is a phenotype that is consistent over time, or if it varies during the individual disease trajectory. The IOS parameters reflecting small airway pathophysiology were significantly correlated comparing the maximal changes during the methacholine/histamine challenge to changes during the EAR, suggesting a consistency in the type of immediate response with fixed major sites of bronchoconstriction, as well as during the LAR, indicating possible associations with factors that promote airway remodeling [40]. Furthermore, oscillometry demonstrates good repeatability over time in asthmatics [58], although we appreciate that larger prospective studies are required to fully characterize this.

Our findings here are clinically relevant, since oscillometry-defined small airways dysfunction is closely associated with poorer symptom control and more frequent severe exacerbations [59]. In essence, oscillometry can be used to predict an individual’s future risk of adverse outcomes and offer clinicians an early opportunity to predict and prevent exacerbations before they occur. It is worth mentioning here that the human small airways express IL4/13, which are in turn associated with airway hyperresponsiveness [60], and may at least in part explain our findings.

### 5.3. Strengths and Limitations

A key strength of the present study is the homogenous study population regarding subject characteristics, methodology and concomitant treatment (i.e., non-smokers, no anti-inflammatory or disease modifying therapies, increased blood eosinophils, HDM allergy and HDM challenge). Recruiting individuals who are not on maintenance anti-inflammatory or asthma-modifying therapeutics offers a unique opportunity to study the LAR in the absence of potential confounders that otherwise may have artificially interfered with its pathophysiology [61]. The absolute mean changes from baseline for FEV_1_, Rrs5-20 and AX exceeded established biological variability values of 150 mL, 0.04 kPa/L/s and 0.39 kPa/L, respectively, indicating clinical relevance and confirming that the methacholine/histamine and the HDM challenges induced significant responses [58]. Equally, however, we acknowledge that our data cannot be extrapolated to smoking asthmatics or those with severe disease.

One limitation is the fact that there is still uncertainty as to whether (any of the) IOS parameters truly reflect small airway physiology. Rrs5-20, for example, is often referred to as peripheral airway resistance based on the idea that Rrs5 reflects the overall airway resistance and Rrs20 the resistance of central airways only, thus indicating that the difference between these two indices would represent the peripheral component. However, there is also a possibility that Rrs5-20 is affected by another process that is believed to be common in asthmatic airways. This phenomenon is referred to as “ventilation heterogeneity”, i.e., the fact that different airways (on the same level) are unevenly affected by bronchoconstriction, yielding a “patchy” pattern rather than a homogeneous one. If one of two airways originating from the same bifurcation is more constricted than the other, the time it takes to fill and empty the volume of air distal to that airway will increase. With lower frequencies of filling and emptying, such as the low frequency of tidal breathing or the lower range of the IOS pulses, time is still sufficient for air to flow in and out of this unit, i.e., the unit of air connected to the constricted airway. As the frequency rises, however, there is an increased possibility that the time required to ventilate this unit exceeds the time allowed by the oscillations, indicating that, with higher frequencies, more of the units distal to constricted airways could be excluded from measurement. Consequently, this would result in a false improvement of the resistance to higher frequencies, e.g., Rrs20, with a subsequent increase in Rrs5-R20. Having said that, previous computational modeling has illustrated that Rrs5-20 and some of the other IOS parameters are closely related to anatomical narrowing of the distal airways [62]. We duly acknowledge that using oscillometry in conjunction with other tests such as imaging or body plethysmography would increase the sensitivity of detecting subtle abnormalities within the respiratory system. However, performing high-resolution CT (HRCT) imaging in asthma patients exposes them to high doses of ionizing radiation, and this limits its use as a follow-up tool. Additionally, oscillometry has been shown to be significantly associated with bronchial wall thickness on HRCT in persistent asthma [5]. Therefore, oscillometry is the most likely tool to be adopted by routine clinical practice due to patient tolerability, safety and good psychometric properties.

### 5.4. Clinical Implications and Future Research

The present study further supports the notion that IOS is sensitive to physiological changes to different bronchoprovocation tests and capable of discriminating between “less severe airway pathophysiology” (MRs to HDM) and “more severe airway pathophysiology” (DRs to HDM). However, before IOS can be fully implemented into clinical practice, there is a need for device-specific reference equations and consensus on what constitutes a positive bronchoprovocation test when defined by IOS parameters [63]. Furthermore, although numerous studies have shown associations among presumed indices of SAD, (peripheral) airway inflammation [5], impaired asthma control and airway remodeling, research on treatment modalities targeting SAD [64] and implications on long-term outcomes on asthma control, lung function (decline), AHR, morbidity and mortality are urgently needed.

As a next step, it would be interesting to assess the relationships between small airway physiology (including oscillometry) and airway inflammation in patients with more severe asthma in the context of more severe type 2 inflammation, AHR and airway remodeling, as well as the interactions with high-dose ICS and other therapeutic options (biologics, AIT, combinations). Another compelling question is whether these relationships still exist in individuals with so-called type 2-low asthma where tezepelumab and macrolides such as azithromycin may play a therapeutic role [65,66]. Exploring additional diagnostic tools such as imaging in relation to allergy response and small airways dysfunction would also deepen these insights.

In conclusion, our findings, with evidence of changes in small airway physiology during allergen-induced LAR and correlations to increased AHR, emphasize the clinical importance of proactive, early treatment of AHR airway inflammation with possible SAD with anti-inflammatory, disease-modifying therapies.

## Figures and Tables

**Table 1:** Subject demographics and baseline measurements.

	Mono-Responders (*n* = 11)	Dual Responders (*n* = 36)	*p* Value
Age (y)	28 (19–51)	26 (18–52)	0.58
Sex, F/M (% female)	5/6 (45%)	22/14 (61%)	0.57
BMI (kg/m^2^)	22.8 (21.7–31.2)	24.4 (17.8–31.7)	0.98
FEV_1_ (L)	3.63 (2.74–4.92) 3.59 (2.69–5.02)	3.32 (2.48–4.87) 3.30 (2.25–4.77)	0.11 0.09
FEV_1_ (% predicted)	94 (83–108) 92 (84–106)	90 (71–125) 90 (68–121)	0.23 0.22
FEV_1_/FVC ratio	0.78 (0.60–0.93) 0.72 (0.65–0.92)	0.76 (0.59–0.90) 0.79 (0.57–0.91)	0.61 0.78
Rrs5 (kPa/L/s)	0.27 (0.17–0.41) 0.30 (0.18–0.45)	0.36 (0.21–0.66) 0.32 (0.20–0.57)	**0.012** 0.16
Rrs5-20 (kPa/L/s)	0.050 (0.000–0.110) 0.040 (0.000–0.130)	0.070 (−0.040–0.180) 0.050 (−0.020–0.210)	0.092 0.55
Rrs5-20/Rrs5 ratio (%)	0.171 (0.000–0.268) 0.133 (0.000–0.323)	0.187 (−0.191–0.405) 0.158 (−0.083–0.429)	0.182 0.87
Xrs5 (kPa/L/s)	−0.090 (−0.160; −0.060) −0.090 (−0.170; 0.030)	−0.120 (−0.230; −0.040) −0.110 (−0.220; −0.040)	0.123 0.22
AX (kPa/L)	0.230 (0.130–0.99) 0.270 (0.040–1.150)	0.611 (0.050–2.51) 0.340 (0.050–2.440)	0.088 0.30
Fres (Hz)	11.03 (9.25–22.67) 12.67 (2.62–20.74)	15.34 (7.30–25.98) 14.90 (7.30–25.73)	0.27 0.42
PBE (cells/µL)	281 (203–504)	320 (150–976)	0.74
Methacholine/histamine PC_20_ (mg/mL)	2.15 (0.53–11.29)	0.84 (0.07–7.51)	**0.036**

All physiology parameters are shown for visit S2 (first row) and visit S3 (second row). Except for sex, all quantitative parameters are shown as medians (minimum–maximum). Statistically significant results in bold font. BMI = body mass index; FEV_1_ = forced expiratory volume in 1 s; FVC = forced vital capacity; Rrs5 = resistance at 5 Hz; Rrs5-20 = frequency dependence of resistance (resistance at 5 Hz minus resistance at 20 Hz); Xrs5 = reactance at 5 Hz; AX = area under reactance curve; Fres = resonant frequency; PBE = peripheral blood eosinophil count; PC_20_ = provocative concentration of methacholine or histamine required to produce a 20% fall in FEV_1_.

**Table 2:** Correlations between the changes in airway physiology parameters during methacholine/histamine challenge and the EAR and LAR post-allergen challenge.

	Meth/Hista vs. 0–3 h Post-HDM	Meth/Hista vs. 3–8 h Post-HDM	0–3 h Post-HDM vs. 3–8 h Post-HDM
FEV_1_	−0.085, *p* = 0.571	0.207, *p* = 0.164	0.275, *p* = 0.062
Rrs5	**0.528, *p* < 0.001**	**0.309, *p* = 0.041**	0.279, *p* = 0.060
Rrs5-20	**0.634, *p* < 0.001**	**0.490, *p* < 0.001**	**0.754, *p* < 0.001**
Rrs5-20/Rrs5	**0.583, *p* < 0.001**	**0.517, *p* < 0.001**	**0.899, *p* < 0.001**
Xrs5	**0.550, *p* = <0.001**	0.060, *p* = 0.703	0.255, *p* = 0.090
AX	**0.644, *p* < 0.001**	**0.379, *p* = 0.011**	**0.582, *p* < 0.001**
Fres	**0.732, *p* < 0.001**	**0.493, *p* < 0.001**	**0.637, *p* < 0.001**

Spearman’s correlation coefficients for the maximal % changes (from post-diluent baseline) for FEV_1_ and IOS parameters, in all subjects (*n* = 47). Statistically significant results in bold font. The maximal % change during meth/hista (=methacholine/histamine) challenge was compared to the maximal % change during the two different phases (EAR and LAR) post-allergen challenge, and the two different phases post-allergen challenge were compared to each other. Significant correlations are marked with bold font. HDM = house dust mite; FEV_1_ = forced expiratory volume in 1 s; Rrs5 = resistance at 5 Hz; Rrs5-20 = frequency dependence of resistance (resistance at 5 Hz minus resistance at 20 Hz); Xrs5 = reactance at 5 Hz; AX = area under reactance curve; Fres = resonant frequency.

## Data Availability

The authors do not have a publicly accessible database where data specifically referring to this research can be found as this is on file at QPS Netherlands and at AMC Netherlands; however the authors have referred to clinicaltrials.gov number where data of the “main study” can be found: NCT03858686.
